# Supplementary material for: A simple, sufficient, and consistent method to score the status of threats and demography of imperiled species
Source: PeerJ. 2016 Jul 14;4:e2230. doi: 10.7717/peerj.2230 (PMC4950543; doi:10.7717/peerj.2230)
Supplement: Article S1 [file peerj-04-2230-s001.docx]

# Text S1. The regulatory framework of listing, reclassification, and recovery

The process from listing to delisting species under the ESA is relatively well defined (Fig. A).

## Pre-listing

FWS evaluates whether a species should be listed or reclassified according to the five-factor threat analysis using “*solely* the best available scientific and commercial information” (50 CFR 424.11). Those factors include (1) present or threatened habitat destruction, modification, or curtailment, (2) over utilization, (3) disease or predation, (4) inadequacy of existing regulations, or (5) other natural or manmade factor affecting its continued existence. Note that demography per se is not one of the factors, even though demography may be affected by the threats. Instead, demography integrated with threats informs the classification as threatened or endangered. Endangered species are those that are “in danger of extinction” throughout all or a significant portion of their range, and threatened species are those that are likely to become endangered in the foreseeable future (Endangered Species Act of 1973, section 3). In reality, the separate analysis of threats and demography is not so clear-cut. A species is proposed for listing in a Federal Register notice if the analysis produces a positive finding, and will be listed if no new information comes to light before the final rule is issued.

## Listed

After listing as either threatened or endangered, the path to delisting starts with recovery planning. Roughly 85% of listed species have recovery plans, which integrate all available information on species’ biology, status, and threats, while outlining recovery objectives and necessary threat-abatement measures (Neel et al. 2012; see also Text S2). After planning, there may be cycles of review, reclassification, or stasis depending on the species’ conservation status.

Every five years by statute, or as work loads allow in reality, FWS reviews a species’ status based on their own data as well as by soliciting data from other agencies and the public. They then produce a “five-year review” in which they synthesize the data and offer recommendations on status changes and future conservation actions. Reviews since the mid-2000s are based on a template that ensures crucial data (if available) is presented for each species. Reviews are available for 80% of listed species (FWS 2012), but a currently unknown number of reviews are out-of-date. Five-year reviews close with recommended status change, if any, and an updated Recovery Priority Number. If a status change is recommended the it will be formalized as a proposed rule in a notice in the Federal Register. After considering feedback from other government agencies and the public, the Service will (typically) change the listing status or (rarely) keep the current status until a subsequent review. FWS has skipped the five-year review in a few instances, instead opting to report the status summary information in the Federal Register notice for the proposed status change.

## Post-listing

Status change recommendations may include a recommendation to delist. To date, 30 species have been delisted (i.e., recovered so that the protections of the Act are no longer needed); 27 species have been “downlisted” (i.e., reclassified from endangered to threatened); ten species have been “uplisted” (i.e., reclassified from threatened to endangered); and 21 species have been declared extinct (FWS 2015). Detailed post-delisting monitoring requirements are part of the delisting final rule.

**Figure A. A simplified overview of the lifecycle of a listing under the Endangered Species Act.** This diagram presents the most-used regulatory decisions and documents of the lifecycle. Alternate decision points and pathways are possible, but are much more rarely used.
