# Supplementary material for: A simple, sufficient, and consistent method to score the status of threats and demography of imperiled species
Source: PeerJ. 2016 Jul 14;4:e2230. doi: 10.7717/peerj.2230 (PMC4950543; doi:10.7717/peerj.2230)
Supplement: Article S2 [file peerj-04-2230-s002.pdf]

Scoring table

Category	Criteria	Score					
		-1	-0.5	0	0.5	1	U
Threats	Most or all threats increased or impossible to address	X					
	Primary threats increased but others eliminated		X				
	Most or all threats continued unabated (no change)			X			
	Primary threats decreased but others increased				X		
	Most or all threats decreased or eliminated					X	
Demography	Most or all populations increased					X	
	Most populations increased but others decreased or eliminated				X		
	Most or all populations remained stable			X			
	Most populations decreased but others increased		X				
	All populations decreased	X					
Either	No information available						X

Examples

Score	Component	Example Species	Example Text	Source
-1	Demographics	Delta smelt	…the *continuing downward trend* in Delta smelt abundance since the significant decline that occurred in 2002…delta smelt abundance indices have *continued to decrease…*a 2005 population viability analysis calculated a 50% likelihood that the species could reach effective extinction within 20 years.	Pg. 2-3
-0.5	Demographics	Southeastern beach mouse	…has been *extirpated* from its southern range…are now found only found in county, state, or Federal lands...Regular surveys of these sites have shown that populations have *remained stable…or decreased* due to the loss of habitat.	Pg. 21
0	Demographics	West Virginia Northern flying squirrel	…species is *persisting throughout its historic range*, with areas of known occupancy occurring much more widespread than at the time of listing.	Pg. 20
0.5	Demographics	West Indian manatee	Populations are *stable to increasing* throughout the majority of the species range.	Pg. 34
1	Demographics	Uncompahgr e fritillary butterfly	…the number of confirmed colonies has *increased from 2-11*. Population estimates have *increased* from about 1,000 to somewhere between 3,400 and 23,000.	Pg. 14
-1	Threats	Lesser long-nosed bat	Despite the reduced incidence of some threats identified at listing in the recovery plan, this recovery criterion has not been met because *new threats have been identified* (border issues, wind energy) and roost sites remain vulnerable (pg.9)…threats to roost sites continue, and in fact, have *likely increased* in recent years (pg. 24).	Pgs. 9 & 24
-0.5	Threats	Delta smelt	We found that threats to the Delta smelt *did not...exhibit significant differences* compared with the 2004-5 review. However, we now have strong evidence, not available at the time of our 2004-5 review, that at least some of those factors *are now endangering the species.*	Pg. 2
0	Threats	Arroyo toad	Threats to the arroyo toad *remain basically the same* as when it was listed in 1994.	Pg. 19
0.5	Threats	Black-capped Vireo	...it appears original *threats to the species still exist*, but the magnitude of threats has changed, resulting in *an overall decrease in threat level.*	Pg. 22
1	Threats	California Least Tern	The least tern recovery effort has *ameliorated threats* to the population so that it is *no longer endangered*.	Pg. 22

Background

The administration and monitoring of conservation programs are closely entwined. Administrators charged with conserving imperiled species must do so under budget and personnel constraints (Ferraro & Pattanayak, 2006). At the national and regional scales, these decision makers need to accurately evaluate hundreds or thousands of species based on their conservation status in order to allocate limited resources efficiently and objectively for the greatest conservation benefit (Bottrill et al., 2008; Joseph et al., 2008). At the same time, assessing the effectiveness of large conservation programs is challenging because of the taxonomic breadth of species and the variety of threats they face (see Purvis et al. [2000] for a summary of the many factors affecting extinction risk). Rarely do metrics capture necessary information concisely and consistently across all species. But such metrics are needed for wildlife managers to effectively allocate resources based on a species' current status.

Administrators of large conservation programs need a small number of highly informative and consistent metrics to accurately evaluate the conservation status of each species and conservation programs as a whole. Two fundamental components of conservation status are a species' demography (e.g., population size, range, and structure) and the threats it faces (Goble, 2009; Neel et al., 2012). Separating these factors is crucial because strategies for addressing threats and demographic status can differ greatly, e.g., population augmentation may improve demographic status while threats that will ultimately undo those gains continue unabated (National Marine Fisheries Service, 2010). Ideally, a small number of monitoring metrics would (a) capture the status or change of threats and demography independently, (b) be designed to apply consistently across all or most listed species, and (c) be easy to calculate given existing data, rather than requiring new and expensive monitoring programs. If such monitoring metrics are available, then the effectiveness of conservation programs can be evaluated in part (e.g., by geographic region) or in whole by analyzing the scores for all species under the program. For example, we could answer questions such as, What is the status of threats across all imperiled species covered by a conservation program? What proportion of imperiled species are declining or improving demographically? Are some regions doing better, on average, at addressing the threats to imperiled species than other regions?

The U.S. Fish and Wildlife Service (FWS) reports two possible conservation status metrics for species listed under the U.S. Endangered Species Act (ESA) in their Biennial Report to Congress. The first metric has changed over the years. Until 2010, FWS reported species status using categories including "declining", "improving", "stable", or "unknown." FWS stopped reporting each species' "status" after 2010 because they judged the conclusions were not scientifically rigorous enough (U.S. Fish and Wildlife Service, 2011). Today, FWS reports

recommendations to reclassify a species' legal status that are based on five-year reviews of each species. Recommendations may include uplisting from threatened to endangered, down-listing an endangered species to threatened, de-listing a species, or no status change (see Article S1 for an overview of the ESA listing lifecycle). The second reported metric is the Recovery Priority Number (RPN), which is used to prioritize recovery planning for ESA-listed species. RPNs are based on the immediacy of threats, recovery potential, taxonomic uniqueness, and conflict with human activities (U.S. Fish and Wildlife Service, 1983). Thus, both metrics contain some information about conservation status and both are used by FWS to allocate resources and make other decisions. But the question remains, are these reported metrics acceptable for monitoring the conservation status of species, or evaluating the effectiveness of the Endangered Species program based on the conservation status of many species?

There are three problems with using the metrics reported by FWS as conservation status metrics. First, a species listed as endangered can't be afforded more protection under the ESA, and neither Congress nor the public receives an early warning if an endangered species has continued to decline. In contrast to IUCN Red List categories that include "critically endangered" and "extinct in the wild" as options before extinction (Rodrigues et al., 2006), the ESA recognizes no classification between "endangered" and "extinct". Second, some changes in either threats or demography may not be sufficient to trigger reclassification, but are still sufficient to warrant the attention of managers during the monitoring and evaluation stages of the recovery and resource allocation process. FWS administrators will be hard-pressed to make informed resource allocation decisions across the endangered species program without simple, sufficient, and consistent metrics of conservation status. Thus, on the first and second counts, recommendations for reclassification have significant shortcomings. Third, although used in conjunction with other information to guide resource allocation (U.S. Fish and Wildlife Service, 2013), RPNs are not sufficient for evaluating species status because they combine many factors, including some that are not conditional on changes of status (e.g., taxonomic uniqueness). Because the conservation status of individual species and groups of species is the ultimate metric by which conservation programs need to be evaluated, neither Congress nor the public can accurately evaluate the effectiveness of the ESA at recovering species using currently reported metrics. Furthermore, some species can "fall through the cracks" of conservation while recovery progress for other species goes unacknowledged. This is not to say that such species receive no attention; biologists and managers in the field may be aware of a species' plight. But regional- or national-level administrators are much less likely to know of these issues, and can't make informed, high-level resource allocation decisions, if unaware of the facts.
