# Supplementary material for: A simple, sufficient, and consistent method to score the status of threats and demography of imperiled species
Source: PeerJ. 2016 Jul 14;4:e2230. doi: 10.7717/peerj.2230 (PMC4950543; doi:10.7717/peerj.2230)
Supplement: Table S1 [file peerj-04-2230-s003.docx]

**Table S1.** Cross-tabulation of taxonomic groups represented in our dataset.

| **Group** | **# improving** | **# declining** | **# no change** | **Taxon totals** |
| --- | --- | --- | --- | --- |
| Crustaceans | 0 | 0 | 1 | 1 |
| Snails | 3 | 1 | 1 | 5 |
| Clams | 0 | 0 | 2 | 2 |
| Insects | 3 | 4 | 3 | 10 |
| Fish | 4 | 3 | 2 | 9 |
| Amphibians | 1 | 0 | 0 | 1 |
| Reptiles | 2 | 1 | 1 | 4 |
| Birds | 8 | 1 | 4 | 13 |
| Mammals | 6 | 0 | 1 | 7 |
| Total | 27 | 10 | 15 |  |
