# Supplementary material for: A simple, sufficient, and consistent method to score the status of threats and demography of imperiled species
Source: PeerJ. 2016 Jul 14;4:e2230. doi: 10.7717/peerj.2230 (PMC4950543; doi:10.7717/peerj.2230)
Supplement: Table S2 [file peerj-04-2230-s004.docx]

**Table S2.** Examples of text from Fish and Wildlife Service five-year reviews that resulted in different scores across the key in Table 1 of the main text.

| **Score** | **Component** | **Example Species** | **Example Text** | **Source** |
| --- | --- | --- | --- | --- |
| -1 | Demographics | Delta smelt | …the *continuing downward trend* in Delta smelt abundance since the significant decline that occurred in 2002…delta smelt abundance indices have *continued to decrease…*a 2005 population viability analysis calculated a 50% likelihood that the species could reach effective extinction within 20 years. | Pg. 2-3 |
| -0.5 | Demographics | Southeastern beach mouse | …has been *extirpated* from its southern range…are now found only found in county, state, or Federal lands...Regular surveys of these sites have shown that populations have *remained stable…or decreased* due to the loss of habitat. | Pg. 21 |
| 0 | Demographics | West Virginia Northern flying squirrel | …species is *persisting throughout its historic range*, with areas of known occupancy occurring much more widespread than at the time of listing. | Pg. 20 |
| 0.5 | Demographics | West Indian manatee | Populations are *stable to increasing* throughout the majority of the species range. | Pg. 34 |
| 1 | Demographics | Uncompahgre fritillary butterfly | …the number of confirmed colonies has *increased from 2-11*. Population estimates have *increased* from about 1,000 to somewhere between 3,400 and 23,000. | Pg. 14 |
|  |  |  |  |  |
| -1 | Threats | Lesser long-nosed bat | Despite the reduced incidence of some threats identified at listing in the recovery plan, this recovery criterion has not been met because *new threats have been identified* (border issues, wind energy) and roost sites remain vulnerable (pg.9)…threats to roost sites continue, and in fact, have *likely increased* in recent years (pg. 24). | Pgs. 9 & 24 |
| -0.5 | Threats | Delta smelt | We found that threats to the Delta smelt *did not...exhibit significant differences* compared with the 2004-5 review. However, we now have strong evidence, not available at the time of our 2004-5 review, that at least some of those factors *are now endangering the species.* | Pg. 2 |
| 0 | Threats | Arroyo toad | Threats to the arroyo toad *remain basically the same* as when it was listed in 1994. | Pg. 19 |
| 0.5 | Threats | Black-capped Vireo | ...it appears original *threats to the species still exist*, but the magnitude of threats has changed, resulting in *an overall decrease in threat level.* | Pg. 22 |
| 1 | Threats | California Least Tern | The least tern recovery effort has *ameliorated threats* to the population so that it is *no longer endangered*. | Pg. 22 |
